# Risk-focused differences in molecular processes implicated in SARS-CoV-2 infection: corollaries in DNA methylation and gene expression

**DOI:** 10.1186/s13072-021-00428-1

**Published:** 2021-12-11

**Authors:** Chaini Konwar, Rebecca Asiimwe, Amy M. Inkster, Sarah M. Merrill, Gian L. Negri, Maria J. Aristizabal, Christopher F. Rider, Julie L. MacIsaac, Christopher Carlsten, Michael S. Kobor

**Affiliations:** 1 BC Children’s Hospital Research Institute (BCCHR), 950 West 28th Avenue, Vancouver, BC V5Z 4H4 Canada; 2 Centre for Molecular Medicine and Therapeutics, Vancouver, BC V6H 0B3 Canada; 3 Canada’s Michael Smith Genome Sciences Centre, BC Cancer, Vancouver, BC V5Z 1L3 Canada; 4 The Department of Ecology and Evolutionary Biology, University of Toronto, Toronto, ON M5S 3B2 Canada; 5 Department of Biology, Queen’ University, Kingston, ON K7L 3N6 Canada; 6 Program in Child and Brain Development, CIFAR, MaRS Centre, 661 University Ave, Toronto, ON M5G 1M1 Canada; 7 The Department of Medical Genetics, University of British Columbia, Vancouver, BC V6T 1Z3 Canada; 8 The Department of Respiratory Medicine, University of British Columbia, Vancouver, BC V5Z 1M9 Canada

**Keywords:** COVID-19, DNA methylation, Gene expression, Sex, Air pollutants, Respiratory conditions

## Abstract

**Background:**

Understanding the molecular basis of susceptibility factors to the severe acute respiratory syndrome coronavirus 2 (SARS-CoV-2) infection is a global health imperative. It is well-established that males are more likely to acquire SARS-CoV-2 infection and exhibit more severe outcomes. Similarly, exposure to air pollutants and pre-existing respiratory chronic conditions, such as asthma and chronic obstructive respiratory disease (COPD) confer an increased risk to coronavirus disease 2019 (COVID-19).

**Methods:**

We investigated molecular patterns associated with risk factors in 398 candidate genes relevant to COVID-19 biology. To accomplish this, we downloaded DNA methylation and gene expression data sets from publicly available repositories (GEO and GTEx Portal) and utilized data from an empirical controlled human exposure study conducted by our team.

**Results:**

First, we observed sex-biased DNA methylation patterns in autosomal immune genes, such as *NLRP2*, *TLE1*, *GPX1*, and *ARRB2* (FDR < 0.05, magnitude of DNA methylation difference Δ*β* > 0.05). Second, our analysis on the X-linked genes identified sex associated DNA methylation profiles in genes, such as *ACE2*, *CA5B*, and *HS6ST2* (FDR < 0.05, Δ*β* > 0.05). These associations were observed across multiple respiratory tissues (lung, nasal epithelia, airway epithelia, and bronchoalveolar lavage) and in whole blood. Some of these genes, such as *NLRP2* and *CA5B*, also exhibited sex-biased gene expression patterns. In addition, we found differential DNA methylation patterns by COVID-19 status for genes, such as *NLRP2* and *ACE2* in an exploratory analysis of an empirical data set reporting on human COVID-9 infections. Third, we identified modest DNA methylation changes in CpGs associated with *PRIM2* and *TATDN1* (FDR < 0.1, Δ*β* > 0.05) in response to particle-depleted diesel exhaust in bronchoalveolar lavage. Finally, we captured a DNA methylation signature associated with COPD diagnosis in a gene involved in nicotine dependence (*COMT*) (FDR < 0.1, Δ*β* > 0.05).

**Conclusion:**

Our findings on sex differences might be of clinical relevance given that they revealed molecular associations of sex-biased differences in COVID-19. Specifically, our results hinted at a potentially exaggerated immune response in males linked to autosomal genes, such as *NLRP2.* In contrast, our findings at X-linked loci such as *ACE2* suggested a potentially distinct DNA methylation pattern in females that may interact with its mRNA expression and inactivation status. We also found tissue-specific DNA methylation differences in response to particulate exposure potentially capturing a nitrogen dioxide (NO_2_) effect—a contributor to COVID-19 susceptibility. While we identified a molecular signature associated with COPD, all COPD-affected individuals were smokers, which may either reflect an association with the disease, smoking, or may highlight a compounded effect of these two risk factors in COVID-19. Overall, our findings point towards a molecular basis of variation in susceptibility factors that may partly explain disparities in the risk for SARS-CoV-2 infection.

**Supplementary Information:**

The online version contains supplementary material available at 10.1186/s13072-021-00428-1.

## Background

Coronavirus disease 2019 (COVID-19), caused by the severe acute respiratory syndrome coronavirus 2 (SARS-CoV-2), erupted in late 2019 and spread quickly across the globe, culminating in a pandemic claiming more than 5,000,000 lives worldwide as of November 2021. This pandemic has led to an unprecedented burden on health care systems, resource supply chains, and economies worldwide. While populations everywhere are affected by COVID-19, there are inequalities in its impact associated with individual patient characteristics [1–5]. Utilizing data from 17 million case records, a comprehensive study in the United Kingdom demonstrated the association of several patient-level variables with COVID-19 related deaths [3]. Demographic variables including sex, age, ethnicity, and pre-existing respiratory illnesses, such as asthma and chronic obstructive pulmonary disease (COPD), were identified as risk factors for COVID-19 mortality. Smaller studies from the United States and Italy have identified similar demographic variables and additional clinical conditions, such as coronary artery disease and hypertension, as risk factors for mortality in critically ill COVID-19 patients [1, 2].

Among these risk factors, males were independently and consistently found to have increased risk for COVID-19 fatality. COVID-19 is not unique in its sex bias, in fact, the SARS outbreak of 2003 also revealed increased mortality among males compared to females [6]. Despite reports of roughly equal numbers of COVID-19 infections in males and females, the Global Health 50/50 research initiative [7] found increased male mortality in sex-disaggregated data from 38 countries [8], findings corroborated by data from China [9, 10], the United Kingdom [3] and the United States [11]. The results of a European study suggest increased disease incidence in males, particularly those over 60 years, followed by increased rates of hospital admission and fatality in affected males [12]. These findings underscore the importance of investigating biological differences between the sexes and how they may contribute to sex-specific risks associated with COVID-19.

Sex influences many aspects of the innate and adaptive immune responses, including the response against viral infections [13, 14]. In the context of SARS-CoV-2 infection, sex differences in immune responses have been reported, and these differences may reflect baseline disparities in immune responses between sexes [15]. For example, T cell responses in female COVID-19 patients were more abundant and robustly activated compared to male COVID-19 patients [15]. Sex differences in immune response to SARS-CoV-2 may be the result of several molecular underpinnings, including established differences in the effects of epigenetic processes on immune responses [16, 17]. A commonly interrogated epigenetic process is DNA methylation, which involves the addition of a methyl group to the cytosine of the 5'carbon, typically at cytosine–phosphate–guanine (CpG) dinucleotides. This epigenetic mark is changeable by environmental exposures and has the potential to regulate gene expression, although this interaction is dependent on genomic context and temporal stage. Amongst other processes, DNA methylation is inherently associated with sex through X chromosome inactivation (XCI), by which DNA methylation coats and transcriptionally silences one of the female X chromosomes to equalize the dosage of X-linked genes between the sexes. However, at least 15% of X linked genes completely escape the process of XCI and an additional proportion show variable inactivation status between tissues and individuals [18–20]. Therefore, epigenetic silencing of the inactive X chromosome is a complex molecular process which may contribute to the sex bias observed in COVID-19 susceptibility, especially because a large number of immune-related genes are encoded by the X chromosome and thus, may be more highly expressed in females [21]. Furthermore, sex hormones such as testosterone and estradiol also contribute to sex differences. Specifically, testosterone reduces immune cell activity by increasing the production of the immunosuppressive cytokine, IL-10 [22] and has been associated with DNA methylation at several CpG sites [23].

Aside from the sex chromosome, sex differences in DNA methylation patterns on the autosomes have also been identified, indicating the epigenetic consequences of sex reach beyond the sex chromosomes [24]. For example, sex-specific differentially methylated regions were identified in immune cell types, including monocytes, T cells and B cells, with a majority of differentially methylated sites located on the autosomes and enriched in immune-related molecules [25], such as immunoglobulin M and lymphocyte-specific protein 1. Furthermore, aging-related epigenetic changes in immune cell types were different between males and females over 65 years of age, suggesting a plausible role of sex in immune system aging [26], which may contribute to the sex differences observed in immune responses against viral infections.

While the role of sex in influencing susceptibility to SARS-CoV-2 infection is increasingly appreciated [15], there is an additional concern that underlying pre-existing chronic respiratory conditions, such as asthma or COPD, may predispose to COVID-19. Interestingly, inhaled corticosteroids administered to severe asthmatic patients may impact SARS-CoV-2 infection by impairing anti-viral immune responses [27] and delaying viral clearance as previously observed in SARS infection [28]. However, no association was reported between asthma diagnosis or inhaled corticosteroids usage and the risk of hospitalization in COVID-19 infected patients [29]. These conflicting findings necessitate more studies to test whether a consistent association might indeed exist. In the context of COPD, a higher risk of developing a severe SARS-CoV-2 infection was reported and COVID-19 infected males with pre-existing COPD showed increased mortality compared to females [5]. However, epigenetic alterations such as differences in DNA methylation that have been reported in COPD and asthmatic patients and associated with functionally relevant gene expression changes [30, 31] remain currently unexplored in the context of SARS-CoV-2 infection.

In addition to demographic factors and pre-existing chronic conditions, individual responses to the environment may also influence risk to COVID-19 and modulate disease severity. Emerging evidence indicates that long-term exposure to air pollutants, including fine particulate matter, sulphur dioxide, and nitrogen dioxide (NO_2_), may worsen disease severity and increase COVID-19 mortality. Several independent international studies have shown that modest increases in traffic-related air pollution are associated with increased COVID-19 morbidity and mortality [32–35]. Most recently, NO_2_ has been shown to be of particular concern in this regard. There is also extensive literature on the association of DNA methylation patterns and air pollutant exposures [36–38]. Such exposures altered DNA methylation at several CpG sites, a subset of which are associated with functionally relevant gene expression changes [37]. Together these findings suggest the importance of investigating air pollution-related DNA methylation as a potential contributor to differences in COVID-19 susceptibility and infection outcomes.

Here, focusing on the molecular underpinnings of risk factors associated with COVID-19, we tested whether human host genes relevant to SARS-CoV-2 infection exhibited sex differences in DNA methylation and/or gene expression. In addition, we investigated whether the molecular profiles of these candidate genes were altered in pre-existing respiratory conditions, such as asthma and COPD. Finally, we tested whether these genes exhibited DNA methylation differences in response to environmental exposures, such as diesel exhaust, particle-depleted diesel exhaust, and allergens.

## Materials and methods

### Data sets

DNA methylation IDATs for nasal epithelia (GSE101641, GSE104471, GSE65163), airway epithelia (GSE85568, GSE137716), blood (GSE111629, GSE174818), airway and lung parenchymal fibroblasts (GSE111396) were downloaded from a publicly available data repository, *GEO.* Although IDAT files were unavailable for lung samples, we downloaded raw unnormalized methylation intensities from GEO (GSE52401) along with log-transformed normalized gene expression data (GSE65205) for the matched nasal epithelia data set. In addition, normalized lung and whole blood gene expression data was obtained from the GTEx portal (version 8). While the GTEx samples were profiled using RNA-sequencing, gene expression data for nasal epithelia was obtained from the Agilent Human Gene Expression Microarray. DNA methylation for the above-mentioned GEO data sets was quantified using the Illumina Infinium HumanMethylation450 BeadChip platform (450K array).

Utilizing data from a controlled human exposure study, conducted at the Air Pollution Exposure Laboratory (Vancouver, Canada), DNA methylation in non-smoking atopic adult participants was measured in three tissues: bronchoalveolar lavage, nasal epithelia, and bronchial airway epithelia. Participants in this study were each exposed to the following conditions, in a randomised order: filtered air with saline (FA + S), filtered air with allergen (FA + A), diesel exhaust with allergen (DE + A) and particle-depleted diesel exhaust with allergen (PDDE + A) [39]. Each exposure was approximately 2 h in duration followed by a 2 min inhaled allergen challenge. This data set also contained samples from asthmatic patients which allowed us to investigate DNA methylation changes in our chosen candidates in relation to an asthma diagnosis. Furthermore, each exposure was separated by a 4-week washout period.

While samples obtained from the controlled human exposure study and GSE174818 were profiled with the Illumina Infinium Human MethylationEPIC BeadChip platform (850 K array), DNA methylation in all the other data sets mentioned above was quantified with the earlier Illumina Infinium Human Methylation450 BeadChip platform (450 K array). Therefore, to enable cross-cohort comparisons, we subsetted to probes that overlapped between the two platforms. The DNA methylation level for each CpG site measured on the arrays was represented as a β value or as a logit-transformed *β* value (*M*-value) for statistical analyses.

### Candidate gene selection

We used a data-informed candidate gene approach to identify differentially expressed genes and differentially methylated CpG sites associated with sex, air pollutant exposure, and asthma. Using Illumina’s annotation, CpG sites from the 450 K array that mapped to the genes of interest were identified [374 autosomal genes, 24 X linked genes (Additional file 1: Table S1)]. Candidate genes were chosen based on the following criteria:

1. Genes involved in SARS-CoV-2 cell entry:
  *ACE2*, *TMPRSS2*, *ADAM17*, *CTSB, CTSL* [40–44]
2. Genes that exhibited sex-specific mRNA levels after SARS-CoV challenge:
  *IL6*, *CCL2* and *CXCL1* [45]
3. 37 validated genes that showed sex-specific expression in blood associated with influenza infection, sex log fold change |> 0.4| [54]
4. Genes involved in ssRNA viral recognition, including SARS-CoV-2

*TLR7* and *TLR8* [46]

5. 332 high confidence host interacting proteins with SARS-CoV-2 [47]
6. 19 sex-specific genes associated with SARS-CoV-2 [48]

### DNA methylation data preprocessing

Aside from the controlled human exposure study, where the three tissue types were randomized across the chips, data preprocessing was performed independently within all samples of each tissue type. Quality control checks were conducted to identify poorly performing samples in all the data sets (https://github.com/kobor-lab/Public-Scripts/tree/master/COVID-19). Functions in the R *ewastools* package were implemented to evaluate 17 control metrics, such as array staining, extension, hybridization, specificity, target removal and bisulfite conversion. Unsupervised hierarchical clustering on the DNA methylation values of the X and Y chromosomes was used to perform sex checks. The detectOutlier() function in the R *lumi* package was also used to identify outliers. The detectionP() and the beadcount() functions in the R *minfi* package identified samples that showed bad detection *p* values in > 1% of their probes and samples that had > 1% of the probes with < 3 beads contributing to the DNA methylation signal. Within tissue sample-to-sample correlation was also performed to assess sample quality.

Subsequently, background correction was performed using preprocessNoob() in the R *minfi* package followed by beta mixture quantile dilation (BMIQ) normalization using the R *wateRmelon* package to correct for probe-type bias on the array. Thereafter, SNP probes, polymorphic probes, cross-hybridizing probes, poorly performing probes (bad detection *p* value > 0.01 in 5% of samples), probes with missing bead count (< 3 beads) were eliminated. Batch effects were corrected using ComBat() in the R *sva* package. XY probes were subset from normalized, batch corrected data sets to perform subsequent analyses on the X-linked genes. For the controlled human exposure data set, samples from all three tissues (bronchoalveolar lavage, bronchial airway epithelia, and nasal epithelia) were preprocessed together and analogous sample quality checks, probe filtering, normalization, and batch correction methods were utilized as described above.

### Gene expression analysis

Normalized gene expression data for nasal epithelia, lung and whole blood was obtained from GEO and the GTEx portal. Male–female differences in gene expression were assessed using a Welch *t* test followed by FDR multiple test correction. FDR adjusted *p* values < 0.05 were considered significant.

### DNA methylation analysis: autosomal loci

To investigate sex associated DNA methylation differences in our candidate genes, we first focused on the infection-relevant respiratory tissues (lung, nasal epithelia, and airway epithelia) and applied a linear model on *M* values for each CpG site using the R *limma* package. Sex was included as the main effect and age and disease status as the covariates. To account for multiple tests, *p* values were adjusted using the FDR method. DNA methylation differences (Δβ) between the sexes was then calculated by subtracting the average *β* of females from the average β of males on an individual CpG site basis. Differentially methylated CpG sites were identified based on statistical (FDR < 0.05) and biological thresholds (Δ*β* > 0.05). We also investigated the sex-specific DNA methylation profiles of these CpG sites in a blood data set.

Next, we explored the controlled human exposure data set to identify if there were DNA methylation differences associated with asthma diagnosis or in response to an allergen and/or air pollutant exposure. To account for within-sample correlation, we fitted a linear mixed effect model using the R *lme4* package. Participant identity was considered a random effect, whereas exposure, sex, asthma, and age were treated as fixed effects. First, we investigated whether altered DNA methylation patterns were observed in asthmatic patients. Second, we sought to determine whether the candidate CpG sites showed exposure-specific (DE + A, PDDE + A, FA + A) DNA methylation differences compared to the control group (FA + S). Resulting *p* values were obtained using likelihood ratio tests of the full model compared against the model without the main effect in question. Tukey’s test using the R *multcomp* package was performed to identify which specific exposure group showed a significant difference compared to the controls.

### X chromosome inactivation and DNA methylation analysis: X chromosomal loci

X-linked β values in females obtained from the 450 K/850 K arrays represent the composite DNA methylation signal of the female active and the inactive X chromosomes. Therefore, we used a previously published [49] method to estimate DNA methylation specific to the female inactive X. As described in Cotton et al. [49] this method enabled approximation of β values at each X-linked CpG site for all female samples; these values were rounded to fall within the range of 0–1. To evaluate the inactivation status of the 24 X-linked candidates, the tissue data sets were filtered to only include 271 CpG sites associated with these genes in the Illumina annotation. Differential DNA methylation between the male X and female inactive X at these CpG sites was evaluated by linear modelling as performed for the autosomal CpG sites. Δβ was calculated by subtracting average male X β from the average female inactive X β at each CpG site. *p* values were adjusted using the FDR method, significance was defined as FDR < 0.05 and Δ*β* > 0.10.

X chromosome inactivation status could be reliably estimated for genes that had high (HC) or intermediate (IC) density CpG island promoters [50]. Genes were classified as escaping XCI if CpG sites in their HC or IC promoters showed average *β* < 15% in both males and females, had overlapping ranges of *β* values in both sexes, or if the *β* value ranges did not overlap, the absolute Δ*β* between the sexes was < 10%. Genes were classified as being subject to/silenced by XCI if CpG island promoter probes exhibited Δ*β* > 10% between the sexes and were significantly differentially methylated (FDR < 0.05) [50]. For the 15 X-linked candidate genes without an HC or IC promoter, XCI status could not be confidently predicted. In these cases, linear modelling was conducted to identify differential DNA methylation between the male X and female inactive X, and the DNA methylation results were compared to differential expression results to yield insights into possible DNA methylation–expression relationships. CpG sites mapping to these genes were considered significantly differentially methylated by sex if they satisfied the criteria of FDR < 0.05 and Δ*β* > 10%.

## Results

**Fig 1.**
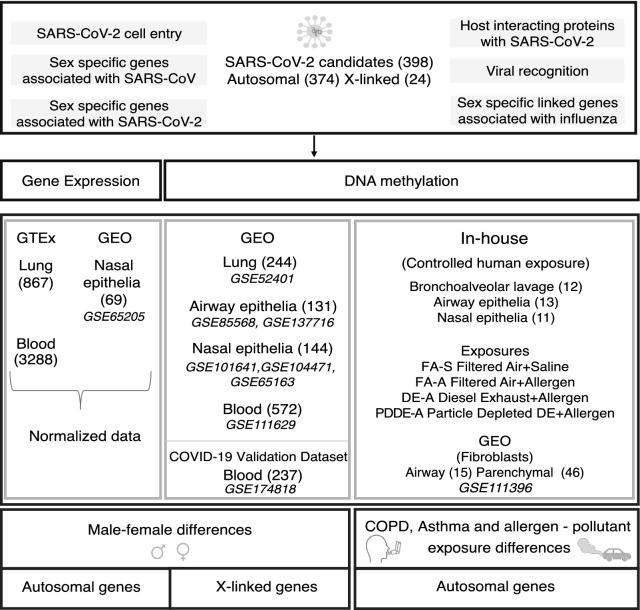
Schematic representation of the methodological approach to the study

Using biologically informed selection criteria (see “Materials and methods” section), we identified 374 autosomal and 24 X-linked candidate genes relevant to COVID-19 biology (Additional file 1: Table S1) and examined if they showed molecular differences in DNA methylation and gene expression related to known COVID-19 risk factors: sex, asthma, COPD and air pollutant exposures (Fig. 1). We downloaded DNA methylation and gene expression data sets from publicly available data repositories, Gene Expression Omnibus (*GEO)* and the Genotype-Tissue Expression *(GTEx)* portal*.* In addition, we utilized DNA methylation data from a controlled human exposure study, using a cross-over design performed at the Air Pollution Exposure Laboratory, Vancouver, Canada (https://pollutionlab.com/).

## Consistent male–female differences in gene expression were observed at SARS-CoV-2 relevant genes

Given that sex influences the transcriptional response to viral infection [51] and is a strong risk factor for COVID-19, we tested whether the genes we selected to be relevant for COVID-19 biology showed male–female differences in mRNA levels in tissues relevant to SARS-CoV-2 infection. To this end, we downloaded normalized lung (596 M, 271F) and whole blood (2166 M, 1122F) gene expression data from GTEx as well as a nasal epithelia mRNA data set (34 M, 35F) from GEO (GSE65205) to assess male–female differences in gene expression at SARS-CoV-2 relevant genes. Mirroring the natural course of SARS-CoV-2 infection in our bioinformatic analysis, we first investigated molecular differences in tissues of the respiratory system: lung and nasal epithelia and then tested for concordance in blood, a tissue highly relevant to COVID-19 biology due to its central role in mediating host immune responses.

### Autosomal loci

**Fig. 2:**
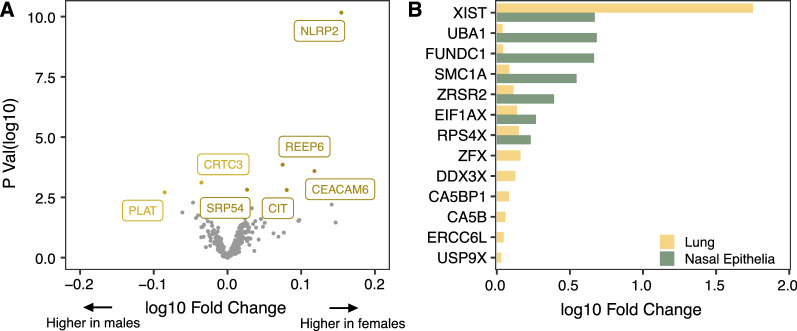
Male–female differences in expression observed in autosomal and X-linked genes. **A** Volcano plot of the differential expression analysis between males and females in autosomal genes (lung). For each gene, *p* values obtained from the Welch *t* tests are plotted on the *y axis*, and the log fold change in gene expression between sexes is plotted on the *x axis*. Genes that met the statistical cutoff of FDR < 0.05 are labeled. Among the five statistically significant genes (*NLRP2, REEP6, CEACAM6, CIT, and SRP54*) which showed decreased expression in males compared to the females, *NLRP2* exhibited the highest change in expression. **B** Bar plot of log fold changes for X-linked genes which were differentially expressed between the sexes in the nasal epithelia and lung. An overlap of five sex-specific genes was observed between the two tissues, of which, *XIST* showed the highest log fold change in expression in females compared to males

Using the R *stats* package, Welch’s *t* tests were conducted on normalized expression values of each candidate gene to identify genes differentially expressed by sex. The Benjamini–Hochberg false discovery rate (FDR) method was used to correct for multiple tests. Using an FDR threshold of < 0.05, we found seven autosomal genes (*NLRP2, REEP6, CEACAM6, CIT, CRTC3, SRP54,* and *PLAT*) in the lung data set that showed significant differences in gene expression between sexes (Fig. 2A, Additional file 1: Table S2). Five out of the seven genes (*NLRP2, REEP6, CEACAM6, CIT,* and *SRP54*) showed decreased expression in males compared to females. Owing to a relatively small sample size in the nasal epithelia data set, we adopted a more lenient FDR threshold of < 0.20 to identify significantly differentially expressed genes associated with sex. Based on this cutoff, 13 autosomal genes displayed male–female expression differences in nasal epithelia (Additional file 1: Table S2), three of which also showed sex-biased expression in the lung. These three genes (*NLRP2, REEP6,* and *CEACAM6*) showed the same direction of sex-biased expression in nasal epithelia and lung samples. Furthermore, male–female expression differences in *NLRP2* were also validated in blood (Additional file 2: Fig. S1) highlighting that *NLRP2* displayed a consistent sex-biased expression pattern across multiple tissues relevant in COVID-19 biology.

### X-linked loci

We applied similar FDR thresholds (< 0.05 for lung; < 0.2 for nasal epithelia) to the 24 X-linked genes and identified 13 X-linked genes differentially expressed by sex in lung, and seven of these genes also had significant sex-biased gene expression in the nasal epithelia (Fig. 2B, Additional file 1: Table S3). These seven genes (*XIST, SMC1A, ZRSR2, EIF1AX, FUNDC1, UBA1,* and *RPS4X*) showed the same direction of male–female differences in both lung and nasal epithelia. Among these, *XIST*, a long non-coding RNA, showed the largest fold change in gene expression by sex in both tissues (lung and nasal epithelia) and in blood, with females showing increased expression compared to males (Additional file 2: Fig. S2).

## Consistent male–female differences in DNA methylation were observed at SARS-CoV-2 relevant genes

Considering that infection induces functional changes in the host DNA methylome [52] and activation of immune responses is influenced by DNA methylation [53], we first investigated DNA methylation differences in the context of sex within our 398 SARS-CoV-2 candidate genes, in three infection-relevant tissues of the respiratory system: nasal epithelia (78M, 66F) (GSE101641, GSE104471, GSE65163), lung (36F, 208M) (GSE52401), and airway epithelia (45M, 86F) (GSE85568, GSE137716). We validated these findings in our smaller unique controlled human exposure data set that comprises of: bronchoalveolar lavage (7M, 5M), nasal epithelia (5M, 6F), and bronchial airway epithelia (7M, 6F). Consistent with our overall strategy, and the central role of blood in the immune response to viral infections, we also tested for concordance of the findings in an independent blood data set (125M, 112F) (GSE111629).

### Autosomal loci

**Fig. 3:**
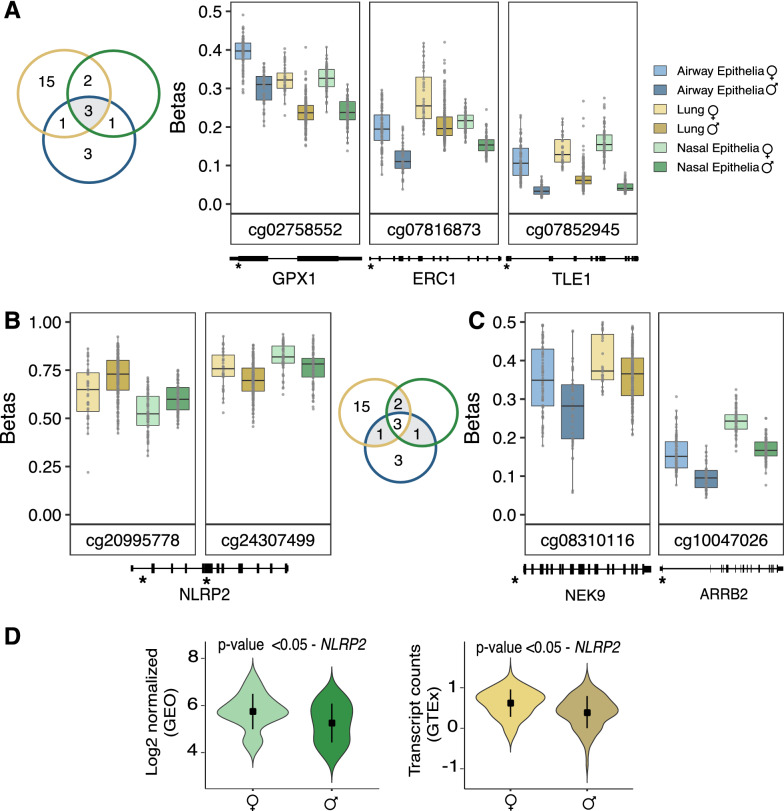
Male–female differences in DNA methylation and gene expression observed in autosomal genes. In the boxplots, unadjusted DNA methylation values (β) were plotted on the *y* axis against the CpG sites on the *x axis*, and genomic positions of the CpG sites were plotted below the respective plots. The overlap of CpG sites across the tissues is shown in the Venn diagrams. In the violin plots, expression was quantified as log2 normalized values for nasal epithelia (GEO) and for the lung data set (GTEx), expression was measured as transcript counts. **A** Boxplots of the three robust CpG sites (cg02758552, cg07816873, cg07852945) which were differentially methylated by sex across the infection relevant tissues. Specifically, at these CpG sites, males exhibited a decreased DNA methylation profile compared to the females. **B**, **C** Boxplots of CpG sites that showed a similar male–female differential DNA methylation pattern in at least two of the infection relevant tissues. **D** Violin plots of *NLRP2* expression differences between males and females in nasal epithelia and lung, respectively

Using the R *limma* package, we applied a linear model on *M* values (logit-transformed *β* value) for each CpG site associated with the 374 candidate autosomal genes. Resulting *p* values were adjusted using the Benjamini–Hochberg FDR method. The magnitude of DNA methylation difference (Δ*β*) between the sexes was calculated by subtracting the average β of females from the average *β* of males per CpG site. Based on statistical (FDR < 0.05) and biological (Δ*β* > 0.05) thresholds, we identified multiple CpG sites that showed male–female differences in DNA methylation in the three infection-relevant respiratory tissues (Additional file 1: Table S4), many of which showed consistent male–female differences across several tissues. Importantly, three CpG sites representing *GPX1*, *ERC1* and *TLE1* genes were differentially methylated by sex in all three respiratory tissues (nasal epithelia, lung, and airway epithelia) with males showing significantly less DNA methylation compared to females (Fig. 3A). We also found similar sex-biased differential DNA methylation patterns in the smaller controlled human exposure data set (bronchoalveolar lavage, nasal epithelia, and bronchial airway epithelia) (Additional file 2: Fig. S3). This DNA methylation pattern was consistent in an independent blood data set as well (Additional file 2: Fig. S4).

CpG sites associated with *NLRP2, NEK9,* and *ARRB2* exhibited similar differential DNA methylation patterns by sex in at least two of the infection-relevant respiratory tissues (FDR < 0.05, Δ*β* > 0.05) (Fig. 3B, C). Interestingly, based on genomic location, the two CpG sites (cg20995778-intron; cg24307499-exon) in *NLRP2* showed male–female DNA methylation differences in the opposite direction. Because DNA methylation patterns are associated with gene transcription profiles, we investigated whether the genes associated with the sex-specific differentially methylated CpG sites also showed male–female differences in gene expression. To that end, we took advantage of our gene expression analysis described above and identified *NLRP2* as the only gene which exhibited male–female differences in both expression and DNA methylation in nasal epithelia and lung (Fig. 3D).

Given that fatality and disease severity to COVID-19 is increased in older adults, we stratified our data by age (0–20 years, 20–30 years, 30–40 years, 40–50 years, 50 + years) and tested whether CpG sites showing sex-biased DNA methylation also exhibited age associated differences. Because information on age was not available for the lung data set, this analysis was performed only on nasal epithelia and airway epithelia. While the distribution of samples across different age groups was not uniform (Additional file 2: Fig. S5), the CpG sites (*GPX1*, *ERC1*, and *TLE1*) which consistently displayed a sex-biased differential DNA methylation pattern across all the investigated tissues also exhibited similar male–female differences in DNA methylation across all age groups (Additional file 2: Fig. S6).

### X-linked loci

DNA methylation β values of the X-linked CpG sites in females represent the composite DNA methylation signal that captures both the female active (lowly methylated) and the inactive X chromosomes (highly methylated), so in females X-linked CpG sites measured by array have higher methylation beta values than males [50]. Therefore, to compare male and female X-linked DNA methylation measured by the array requires alternate methods, as direct male–female comparisons identify widespread higher female DNA methylation across the X chromosome, due to X chromosome inactivation [54]. Sex-specific phenotypes related to the X chromosome can arise when genes escape from X inactivation and are, therefore, expressed biallelically in females [55], one such alternate method of investigation is the use of X-linked promoter DNA methylation to predict the X chromosome inactivation status in females [50, 54]. To estimate DNA methylation levels specific to the female inactive X chromosome, we evaluated the XCI status (as described in the methods section) [49] for 15 of the 24 candidate X-linked genes which were located proximal to high-density (HC) or intermediate-density (IC) CpG island promoters, as required for accurate X inactivation status estimation [54]. Differential DNA methylation analysis was conducted for all CpG sites associated with these genes (Additional file 1: Table S5).

**Fig. 4:**
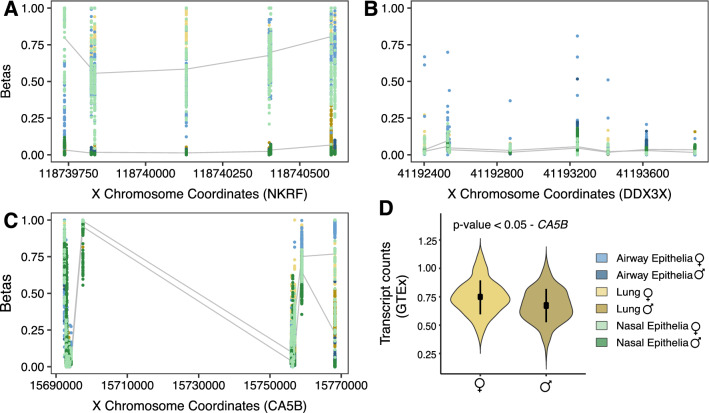
Male–female differences in DNA methylation and gene expression in X-linked genes. Scatter plot of DNA methylation values (β) on the *y axis* and genomic distances to the most proximal transcription start site are on the *x axis*. The lines indicate the average DNA methylation values (β) in males and on the female inactive X across the infection relevant tissues. **A** *NKRF*, an example of a gene subjected to X chromosome inactivation showed significantly higher DNA methylation levels in females compared to males. **B** *DDX3X*, an example of a gene with a high-density CpG island promoter was predicted to escape X chromosome inactivation and thus, the inactive X promoter DNA methylation levels in females were as low as male X promoter DNA methylation levels. **C** *CA5B*, an example of a gene that did not possess a high-density or an intermediate-density CpG island promoter and hence, the X chromosome inactivation status could not be reliably estimated. However, linear modeling between the male X and female inactive X showed that the female inactive X was more methylated across the 5’ untranslated region and along the gene body. **D** Correspondingly, *CA5B* gene demonstrated significantly higher expression in females relative to males.

X-linked genes that show higher promoter DNA methylation on the female inactive X compared to the male X tend to be effectively silenced by, or “subject” to, XCI, whereas genes with similar inactive X promoter DNA methylation in females compared to male X are likely to escape from XCI and be expressed from the inactive X. Of the HC or IC promoter genes inspected, the DNA methylation profiles of six genes (*APT6AP1, ERCC6L, GRIPAP1, POLA1, RBM41,* and *NKRF*) suggested that they would be subject to silencing by XCI in the three COVID-19 infection-relevant tissues (Exemplified by *NKRF* in Fig. 4A), which was replicated in blood as well. These six genes have been previously reported to be subject to XCI in blood and cell culture experiments [56]. The remaining nine genes with an HC or IC promoter were predicted to escape XCI and are likely expressed from both the inactive and active X, based on low inactive X promoter DNA methylation similar to male X promoter DNA methylation levels. These genes were found to escape XCI in the three infection-relevant tissues, and showing concordance in blood samples: *CA5BP, E1F1AX, FUNDC1, RPS4X, UBA1, USP9X, ZFX,* and *ZRSR2,* and *DDX3X* (Exemplified by *DDX3X* in Fig. 4B).

Genes that did not have HC or IC promoters were evaluated for differential DNA methylation between the male X and female inactive X chromosome by linear modelling. Of the nine genes considered, eight contained CpG sites that demonstrated sex associated differences at multiple CpG sites across the gene body and/or associated regulatory regions in at least one tissue. Five of these genes contained 27 differentially methylated CpG sites across the three infection-relevant respiratory tissues. Differential DNA methylation at the same CpG sites validated in blood as well: *ACE2, CA5B, HS6ST2, TLR8,* and *XIST*. Two of these, including *CA5B* and *HS6ST2* also showed male–female differences in gene expression in the GTEx data (Exemplified by *CA5B* in Fig. 4C, D). While *ACE2*, the cell entry receptor for SARS-CoV-2 did not show sex-biased mRNA expression differences, we observed increased DNA methylation on the female inactive X when compared to the male X at multiple CpG sites across the respiratory tissues and blood. An X inactivation evaluation was not made for *ACE2* with DNA methylation data as it lacked a suitable HC/IC promoter for this method, and therefore, sex-biased DNA methylation should be interpreted for this gene in the context of gene expression evidence, which suggested it was not differentially expressed in these tissues in relation to sex.

### Interrogation of SARS-CoV-2 relevant gene DNA methylation revealed an association with chronic respiratory diagnosis in COMT

Because pre-existing chronic respiratory conditions, such as asthma and COPD, may affect COVID-19 susceptibility and mortality [3], we tested whether altered DNA methylation patterns were observed at COVID-19 relevant genes in asthmatic and COPD patients. Utilizing the information on asthma diagnosis, we first investigated the controlled human exposure data set and examined the relationship between DNA methylation and asthma in three infection-relevant respiratory tissues (bronchoalveolar lavage: *n* = 12, nasal epithelia: *n* = 11, and bronchial airway epithelia: *n* = 13). A similar study design utilized for the controlled human exposure data set is described by the authors in a previous publication [39]. We fitted linear mixed effect models on *M* values using the R *lme4* package, with participant id included as a random effect and exposure, sex, and age considered as fixed effects. Perhaps, owing to the relatively low prevalence rate of asthma in this data set, we did not observe significant DNA methylation differences associated with an asthma diagnosis at the candidate CpG sites.

**Fig. 5:**
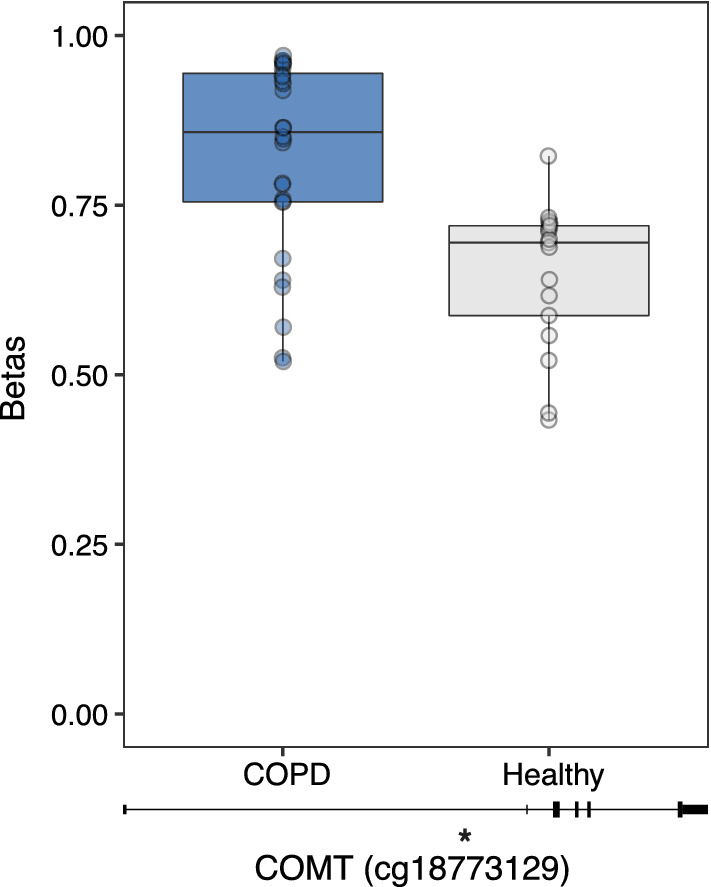
Difference in DNA methylation between COPD patients and non-COPD controls in lung parenchymal fibroblasts. For *COMT* associated CpG site (cg18773129), unadjusted DNA methylation values (β) were plotted on the *y* axis and the genomic location of the CpG site is shown below the boxplot

Using a publicly available GEO data set (GSE111396), we next tested whether CpG sites associated with the COVID-19 candidate genes were altered in COPD patients compared to controls in airway (*n* = 15) and lung parenchymal fibroblasts (*n* = 46). We found no differentially methylated CpG sites associated with COPD diagnosis in airway fibroblasts; however, one CpG site (cg18773129; *COMT*) in parenchymal fibroblasts showed increased DNA methylation in COPD patients compared to healthy controls (FDR < 0.2, Δ*β* > 0.05) (Fig. 5).

### DNA methylation patterns were associated with environmental exposures in bronchoalveolar lavage

Given the emerging literature on exposure to environmental pollutants and SARS-CoV-2 infection [32, 33], we analyzed DNA methylation profiles of our candidate genes in response to air pollutant exposures in our controlled human exposure data set (bronchoalveolar lavage, nasal epithelia, and bronchial airway epithelia). Individuals in this data set were exposed to each of these four conditions, in a randomised order: filtered air with saline (FA + S), filtered air with allergen (FA + A), diesel exhaust with allergen (DE + A) and particle-depleted diesel exhaust with allergen (PDDE + A) [39]. Linear mixed effect models using the R *lme4* package were fitted on individual CpG sites associated with our 374 autosomal candidate genes. Participant id was included as a random effect and exposure, sex, asthma, and age were considered as the fixed effects.

**Fig. 6:**
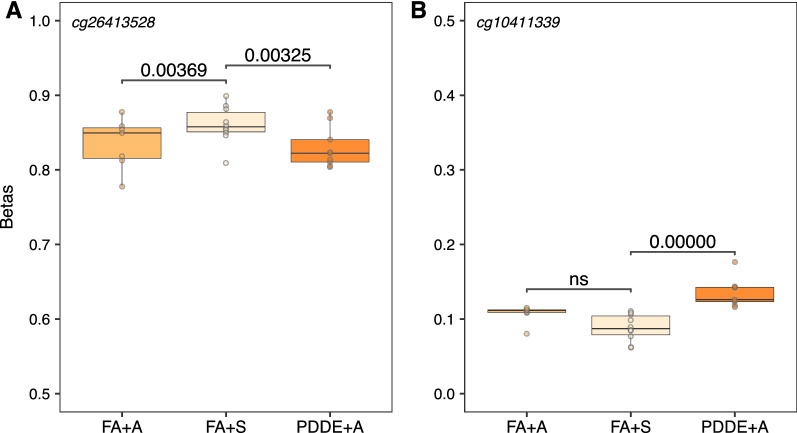
DNA methylation patterns in response to environmental exposure to pollutants in bronchoalveolar lavage. Box plots of unadjusted DNA methylation values (β) on the *y axis* against the exposure groups on the *x axis*. **A** At cg26413528, both the allergen exposed group (FA + A) and the particle-depleted diesel exhaust (PDDE + A) group showed a decreased DNA methylation profile compared to the control group (corrected *p* value < 0.05). **B** At cg10411339, only the particle-depleted diesel exhaust (PDDE + A) group showed an increased DNA methylation profile compared to the control group (corrected *p* value < 0.05)

For the autosomal CpG sites, we found no significant DNA methylation differences in response to pollutant and allergen exposures in nasal and bronchial airway epithelia; however, two CpG sites (cg26413528, cg10411339) in bronchoalveolar lavage showed altered DNA methylation profiles in the PDDE + A exposed group compared to the FA + S controls (FDR < 0.1; Δ*β* > 0.025) (Additional file 1: Table S6). Since individuals were subjected to a 2 min inhaled allergen challenge after being exposed to a pollutant for 2 h, it was important to determine whether the observed DNA methylation patterns were reflective of the pollutant or the allergen. We performed Tukey tests using R *multcomp* package and identified the specific exposure group/s which showed DNA methylation differences at the two CpG sites mentioned above. Significant DNA methylation differences between the PDDE + A exposed and the FA + S controls were observed for both CpG sites (FDR < 0.05), although, the magnitude of DNA methylation difference was relatively small (Δ*β* < 0.05), perhaps not surprising given the short exposure times employed in these experiments (Fig. 6). Specifically, cg26413528 (*PRIM2*) showed decreased DNA methylation in the FA + A group compared to the controls; the same DNA methylation pattern was observed in the PDDE + A individuals (Fig. 6A). However, at cg10411339 (*TATDN1*), no significant DNA methylation differences between the allergen exposed group (FA + A) and the control group (FA + S) were noted (Fig. 6B). In addition, given our sample size in the controlled human exposure data set, we could not perform reliably powered sex-stratified analyses on the X-linked CpG sites.

### DNA methylation sex differences at COVID-19 relevant genes recapitulate in SARS-CoV-2 infected blood samples

To begin exploring whether our computational predictions were reflective of the real-world data related to SARS-CoV-2 infections, we took advantage of a DNA methylation COVID-19 data set (GSE174818) that became publicly available during the revision of our manuscript. The nature of this data set allowed us to examine the concordance between the CpG sites that we identified as differentially methylated in association with sex, and their empirical patterns in the context of actual SARS-CoV-2 infection. Focusing on our set of CpG sites identified as being differentially methylated by sex in at least two of the infection-relevant respiratory tissues and further validated in our large control blood data set (n-237), we performed a sex-stratified analysis in COVID-19 positive cases versus COVID-19 negative controls. Using similar statistical approaches as the ones used in our previously described data sets, we first validated the sex-specific patterns of the identified sex-biased CpG sites from our previous analyses. Second, we tested for potential associations with SARS-CoV-2 infection status in both autosomal regions and on sex chromosomes.

**Fig. 7:**
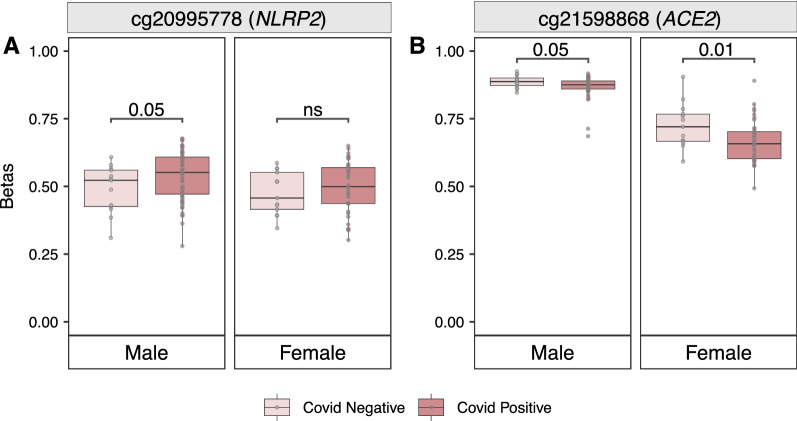
Sex differences in DNA methylation stratified by COVID-19 status. Box plots of Unadjusted DNA methylation values (*β*) plotted on the *y* axis and the box plots are colored by COVID-19 status. **A** Box plots of cg20995778 in *NLRP2* which is differentially methylated by COVID-19 status in only males. **B** Box plots of cg21598868 in *ACE2* differentially methylated by COVID-19 status in both males and females; and females (Δ*β* = 0.061) show a larger effect size than males (Δ*β* = 0.037)

For autosomal loci, we found that our set of identified sex-biased CpG sites also showed differential DNA methylation patterns by sex in the COVID-19 data set (*p* value 0.05). Furthermore, one CpG site in *NLRP2* (cg20995778; *p* value ≤ 0.05 and Δβ > 0.05) was differentially methylated in males when COVID-19 positive cases were compared to COVID-19 negative controls (Fig. 7A). Females showed the same trend without reaching statistical significance, likely connected to having an overall much smaller biological effect size (Δ*β* < 0.02). Next, when examining X-linked candidate loci, we found that one CpG site in *ACE2* (cg21598868, *p* value ≤ 0.05) was differentially methylated by COVID-19 status in both sexes, although females (Δβ = 0.061), had a larger effect size than males (Δ*β* = 0.037) (Fig. 7B). In addition, in our sex-stratified analysis and at a *p* value ≤ 0.05, cg24735671 (*TLR7,* Δ*β* = 0.043) and cg20981403 (*TLR8,* Δ*β* = 0.036) showed differential DNA methylation by COVID-19 status only in males, whereas cg19782749 (*HS6ST2*, Δ*β* = 0.045) and cg00741717 (*TLR8*, Δ*β* = 0.042) were differentially methylated in females (Additional file 2: Fig. S7).

## Discussion

Understanding the molecular basis of variation in risk factor disparities for SARS-CoV-2 infection may provide valuable information needed to understand biological processes affected during the infection. To gain deductive insights into the disease, we examined gene expression and DNA methylation patterns in disease relevant tissues at biologically informed candidate genes relevant in SARS-CoV-2 infection and provided evidence for molecular differences between the sexes, those with pre-existing respiratory conditions, and in response to air pollutants.. First, we tested sex differences in DNA methylation and gene expression and identified consistent effects at several immune-related genes across multiple tissues. We found that a subset of the sex-biased molecular differences also exhibited differential DNA methylation patterns by COVID-19 status in an empirical human data set, thus supporting the relevance of our computational predictions for DNA methylation patterns associated with actual SARS-COV-2 infection. Second, we identified differential DNA methylation of a CpG loci within the COMT gene in lung parenchymal fibroblasts associated with COPD, a risk factor in COVID-19. Third, our candidate analysis detected environmental exposure-related DNA methylation patterns, which may also contribute to differences in SARS-CoV-2 infected individuals.

In the context of molecular sex differences, *NLRP2* showed sex-specific differences in both DNA methylation and gene expression across multiple infection-relevant tissues. *NLRP2* belongs to the NOD-like receptor family, which functions as a pattern recognition receptor (PRR) to identify pathogen associated molecular patterns in infectious agents. Upon recognition, these PRRs activate inflammatory responses in the host to eliminate the pathogen. *NLRP2* functions as a suppressor of proinflammatory responses by inhibiting type 1 interferon responses and the nuclear factor-κB pathway, a signaling cascade implicated in SARS-CoV-2 infection [57–59]. Thus, NLRP2 participates in a negative feedback loop to suppress a hyperactive immune response, which is often observed in COVID-19 patients [60–62].

While the role of *NLRP2* is not specifically characterized in SARS-CoV-2 infection, *NLRP2* has shown sex-biased expression in influenza infection [63], and another member of the same NOD-like receptor family, *NLRP3*, is involved in inducing pulmonary inflammation in COVID-19 [64]. In general agreement with our findings, a few studies have previously confirmed the sex-specific expression profile of *NLRP2,* with decreased expression observed in males relative to females [65, 66]. Expanding beyond these situations and integrating our computational predictions with empirical data on SARS-COV-2 infection in human whole blood, we found a CpG site in *NLRP2* that was not only differentially methylated in COVID-19 positive males compared to COVID-19 negative males, but also displayed a relatively larger magnitude of DNA methylation difference by COVID-19 status in males relative to females. Based on the observed associations and the ability of *NLRP2* to inhibit proinflammatory responses, it is tempting to speculate that males may be more likely to manifest an NLRP2-mediated hyperactive inflammatory phenotype in response to SARS-CoV-2 infection. Like *NLRP2*, other immune-related genes exhibited similar sex-biased differential DNA methylation patterns, such as *ARRB2*, *TLE1*, and GPX1, also inhibitors of the nuclear factor-κB pathway [67, 68]. Moreover, *TLE1* also regulates several target genes in the Wnt pathway [69], a signaling pathway important for sex differentiation [70, 71] and viral replication [72]. Interestingly, in a recent investigation, members of this pathway were identified as potential biomarkers for prognosis and treatment of SARS-CoV-2 infected patients with acute respiratory distress syndrome [73]. In addition to *TLE1*, a CpG site in the 5’UTR of GPX1, a selenium-dependent antioxidant, which interacts with the main protease of SARS-CoV-2, consistently showed male–female DNA methylation differences across all the tissues investigated in the current study. During a viral infection, such as in the case of SARS-CoV-2, the increased production of reactive oxygen species such as hydrogen peroxide is counterbalanced by antioxidants, namely, GPX1, which catalyzes hydrogen peroxide to water. Of note, hydrogen peroxide has been shown to stimulate nuclear factor-κB signaling pathway [74], which activates several proinflammatory cytokines in the innate immune response network. Collectively, these findings suggested nuclear factor-κB signaling pathway as an additional important pathway in understanding sex differences in COVID-19 susceptibility and severity.

Among the X-linked genes, *ACE2*, the host cell receptor which binds to SARS-CoV-2, showed increased DNA methylation on the female inactive X as compared to the male X. The same direction of sex-biased DNA methylation pattern in *ACE2* was observed in a recent publication [75], although the authors did not specifically investigate the inactive X; together, our results suggested that *ACE2* was more methylated at both female X chromosomes than the male X. Perhaps most reassuringly for the validity of our approach, we also identified cg21598868 in *ACE2* as being differentially methylated by COVID-19 status consistently in both males and females. We note that females displayed a relatively larger methylation difference at this particular CpG site, potentially pointing towards a molecular association that may explain some of the sex-associated differences in SARS-CoV-2 infected individuals. While we did not detect significant differential mRNA expression of *ACE2* between the sexes in our tissues, other studies have, in fact, also reported inconsistent results on the association of *ACE2* expression with sex [76–79]. Some of these inconsistencies may be attributable to differences in tissue type, sample size, age, and population. Intriguingly, in a recent preprint [80], regulatory elements within *ACE2* have altered the expression of neighboring X-linked genes, such as *CA5B*, which in the current study also exhibited sex differences in both DNA methylation and mRNA expression. These findings suggested that ACE2 may regulate transcription of key genes involved in interactions of the host with SARS-CoV-2 and thus, may indirectly contribute to some of the molecular differences between sexes that can influence the risk to COVID-19. Taken together, these molecular sex differences in SARS-CoV-2 candidates suggest sex is an important consideration in COVID-19 research with implications for understanding disease pathophysiology. Although women have been fairly represented in the published randomized-controlled trials of COVID-19, these trials did not perform sex-specific analyses [81], which may have adverse consequences on screening and medical interventions for COVID-19, given that there are male–female differences in molecular processes of genes relevant in COVID-19 biology.

Beyond molecular sex differences, pre-existing respiratory conditions might confer an increased risk to SARS-CoV-2 infection. Utilizing molecular data on two such respiratory conditions, asthma or COPD, we investigated DNA methylation effects associated with an asthma and COPD diagnosis for our set of candidate genes. No detectable DNA methylation differences associated with asthma were observed, indicating either there were no DNA methylation alterations at the candidate CpG sites, or the sample size was too small to detect them. Informatively, a comprehensive study [82] on the prevalence of asthma in COVID-19 patients, with data collected from several countries across the world, pointed towards an unclear association between asthma and COVID-19 susceptibility as well as severity. In contrast, at our moderately stringent analytical threshold, we identified one CpG site that was differentially methylated in COPD patients compared to controls, this CpG site was in COMT, a gene implicated in nicotine dependence and cigarette smoking. Of note, COPD patients in this study were either current or former smokers, whereas the healthy controls were non-smokers. Therefore, DNA methylation patterns observed at this site may either represent a true association with the primary variable “COPD”, with the confounding variable “smoking”, or may even reflect a compounded effect of both known risk variables in COVID-19.

In addition to risk factors such as sex and pre-existing respiratory conditions, exposure to air pollutants correlated with increased susceptibility, severity and COVID-19 mortality [83, 84]. While both population-based and controlled exposure studies have reported the effects of air pollutants on molecular processes [38], the influence of such pollutants on genes relevant to COVID-19 biology have not been extensively studied in the context of DNA methylation. Here, we found altered DNA methylation at two CpG sites associated with exposure to particle-depleted diesel exhaust and allergen. Specifically, cg26413528 showed decreased DNA methylation levels that were observed in both the allergen and the particle-depleted diesel exhaust group and this CpG site has previously been associated with respiratory condition in another independent study [85]. At the other CpG site (cg10411339), we did not observe any significant differences between the allergen exposed and the controls, suggesting that the altered DNA methylation profile likely reflects exposure changes that are only related to particle-depleted diesel exhaust and not the allergen itself. We note that particulate-depleting technologies minimize the adverse effects of air pollutants by filtering PM_2.5_ (particulate matter ≤ 2.5 μm in aerodynamic diameter). However, in the process of particle depletion, the gaseous composition is altered such that an increase in NO_2_ levels is often reported. In this context, it is interesting that exposure to high NO_2_ levels has been recently identified as a potent contributor to COVID-19 infection and also mortality [86, 87]. We thus speculate that the exposure associated DNA methylation patterns observed in the study, which captured differences in NO_2_ levels, may underlie molecular variation in COVID-19 susceptibility [39]. Notably, these findings were observed only in bronchoalveolar lavage, a tissue not routinely collected and tested for COVID-19, although the diagnostic accuracy of bronchoalveolar lavage specimens in detecting SARS-COV-2 infection is high [88, 89]. In addition to tissue-specific DNA methylation, the magnitude of DNA methylation changes between exposed and non-exposed groups were relatively small, which is expected, given that the individuals in these experiments were subjected to short exposure timeframes. However, these small molecular effects may still represent meaningful changes that may explain some of the differences following SARS-CoV-2 infection [90]. In addition, it has been documented that molecular sex differences in both DNA methylation and gene expression tend to be small to moderate [91, 92], thus generally showing similar magnitude of differences to the ones reported in our associations.

Although the findings presented here offer risk-focused molecular insights into COVID-19, this study has inherent limitations that should be acknowledged. First, the findings described in this study reflect an association of molecular differences in human host genes relevant in COVID-19 and do not establish any causal mechanisms linked with the disease. While in our analysis we did indeed observe some of the sex-biased DNA methylation differences in an empirical COVID-19 data set from blood, it will be imperative to not only expand this to additional human data sets and tissues, but to also determine the functional relevance of these candidates, if any, using model organisms or an in vitro system. Second, our analysis of sex differences in DNA methylation and gene expression patterns relied exclusively on a sex chromosome complement definition of sex; however, we acknowledge both the existence of intersex individuals and the many other aspects of sex, including secondary sex characteristics and fluctuating sex hormone levels [93]. In addition, in our analyses, sex cannot be disentangled from gender identity and gendered experiences, especially in regard to sex and gender differences in COVID-19 outcomes and health care [94, 95] events. Third, except for a few of our candidates, such as *NLRP2* and *CA5B*, we observed unique changes in distinct genes associated with the two molecular processes. It is important to note that the correlation between DNA methylation and gene expression is not straightforward [96, 97] and is dependent on genomic context and temporal stage. Furthermore, genetic variation, which is not measured in this study, impacts both DNA methylation and gene expression; in fact, both DNA methylation and genetic variation may sometimes work in tandem to influence gene expression [98]. Finally, our sample size was relatively small, especially for the controlled human exposure data set and comprised of ethnically less diverse individuals; therefore, these findings might provide an important starting point for confirmatory studies in larger populations with diverse ancestries. Finally, an important limitation of the candidate gene approach adopted in this study is the inability to discover novel associations beyond those genes chosen as putative candidates.

## Conclusion

Together these data provide evidence for the importance of molecular differences at the level of DNA methylation and gene expression in understanding SARS-CoV-2 susceptibility and development, especially regarding molecular sex differences, pre-existing respiratory conditions, and air pollutant exposure. Further investigation into the implications of these differences could be integral for a better understanding of disease susceptibility and outcomes of COVID-19. This work lays a foundation to build upon in our collective effort to combat this worldwide pandemic and improve outcomes for all.

## Supplementary Information

**Additional file 1: Table S1.** List of candidate genes examined in the current study. **Table S2.** Results obtained from the sex-based expression analysis performed on the autosomal genes (segregated by tissues). **Table S3.** Results obtained from the sex-based expression analysis performed on the X-linked genes (segregated by tissues). **Table S4.** Results obtained from the sex-based DNA methylation analysis performed on the autosomal genes (segregated by tissues). **Table S5.** Results obtained from the sex-based DNA methylation analysis performed on the X-linked genes (segregated by tissues). **Table S6.** Results obtained from the exposure-based DNA methylation analysis performed on the autosomal genes (segregated by tissues).

**Additional file 2: Fig S1.** Violin plots of sex-biased gene expression in NLRP2. Expression in blood was quantified as transcript counts (GTEx). **Fig S2.** Violin plots of sex-biased gene expression in XIST. (A)Expression in nasal epithelia was measured as log2 normalized values (GEO). (B)Expression in lung was quantified as transcript counts (GTEx). (C)Expression in blood was quantified as transcript counts (GTEx). **Fig S3.** Box plots of the three validated CpG sites (GPX1, ERC1 and TLE1) differentially methylated by sex in three tissues from the repeated exposure data set. Unadjusted DNA methylation values (β) were plotted on the *y* axis with CpG sites on the *x* axis. Genomic positions of the CpG sites are indicated below the respective plots. **Fig S4.** Box plots of the three validated CpG sites (GPX1, ERC1 and TLE1) differentially methylated by sex in blood. Unadjusted DNA methylation values (β) were plotted on the *y* axis against the CpG sites on the *x* axis, with genomic locations of the CpG sites plotted below the respective plots. **Fig S5.** Bar plots of age distributions in airway epithelia and nasal epithelia. Age group in years is indicated on the *y* axis while the *x* axis represents the number of males and females. **Fig S6.** Box plots of the three validated CpG sites (GPX1, ERC1 and TLE1) differentially methylated by sex across the age groups in airway epithelia and nasal epithelia. Unadjusted DNA methylation values (β) were plotted on the *x* axis with age groups in years on the *y* axis. Genomic positions of the CpG sites are represented below the respective plots. **Fig S7.** Box plots of the CpG sites that are differentially methylated by COVID-19 status in males (A) and in females (B). Unadjusted DNA methylation values (β) were plotted on the *y* axis and the box plots are colored by COVID-19 status.

## Data Availability

Accession numbers for the publicly available data sets utilized in the study are mentioned in the methods section. Data needed to evaluate the paper are either presented in the manuscript and/or the Supplementary Materials. The controlled human exposure data set utilized in the current study is available on the KoborLab Github repository (https://github.com/kobor-lab/Data). Additional relevant data, if required is available on request. Data preprocessing R scripts are available on the KoborLab Github repository (https://github.com/kobor-lab/Public-Scripts/tree/master/COVID-19).

## Abbreviations

BMIQ: Beta mixture quantile dilation
COPD: Chronic obstructive pulmonary disease
COVID-19: Coronavirus disease 2019
DE + A: Diesel exhaust with allergen
FA + A: Filtered air with allergen
FA + S: Filtered air with saline
FDR: False discovery rate
GEO: Gene Expression Omnibus
GTEx: Genotype-Tissue Expression
HC: High-density
IC: Intermediate-density
NO2: Nitrogen dioxide
PDDE + A: Particle-depleted diesel exhaust with allergen
PM2.5: Particulate matter ≤ 2.5 μm in aerodynamic diameter
PRR: Pattern recognition receptor
SARS-CoV-2: Severe acute respiratory syndrome coronavirus 2
XCI: X chromosome inactivation
850 K array: Illumina Infinium Human MethylationEPIC BeadChip platform
450 K array: Illumina Infinium Human Methylation450 BeadChip platform
