# Supplementary material for: Risk-focused differences in molecular processes implicated in SARS-CoV-2 infection: corollaries in DNA methylation and gene expression
Source: Epigenetics Chromatin. 2021 Dec 11;14:54. doi: 10.1186/s13072-021-00428-1 (PMC8665859; doi:10.1186/s13072-021-00428-1)
Supplement: Supplementary file 2 — Additional file 2: Fig S1. Violin plots of sex-biased gene expression in NLRP2. Expression in blood was quantified as transcript counts (GTEx). Fig S2. Violin plots of sex-biased gene expression in XIST. (A)Expression in nasal epithelia was measured as log2 normalized values (GEO). (B)Expression in lung was quantified as transcript counts (GTEx). (C)Expression in blood was quantified as transcript counts (GTEx). Fig S3. Box plots of the three validated CpG sites (GPX1, ERC1 and TLE1) differentially methylated by sex in three tissues from the repeated exposure data set. Unadjusted DNA methylation values (β) were plotted on the y axis with CpG sites on the x axis. Genomic positions of the CpG sites are indicated below the respective plots. Fig S4. Box plots of the three validated CpG sites (GPX1, ERC1 and TLE1) differentially methylated by sex in blood. Unadjusted DNA methylation values (β) were plotted on the y axis against the CpG sites on the x axis, with genomic locations of the CpG sites plotted below the respective plots. Fig S5. Bar plots of age distributions in airway epithelia and nasal epithelia. Age group in years is indicated on the y axis while the x axis represents the number of males and females. Fig S6. Box plots of the three validated CpG sites (GPX1, ERC1 and TLE1) differentially methylated by sex across the age groups in airway epithelia and nasal epithelia. Unadjusted DNA methylation values (β) were plotted on the x axis with age groups in years on the y axis. Genomic positions of the CpG sites are represented below the respective plots. Fig S7. Box plots of the CpG sites that are differentially methylated by COVID-19 status in males (A) and in females (B). Unadjusted DNA methylation values (β) were plotted on the y axis and the box plots are colored by COVID-19 status. [file 13072_2021_428_MOESM2_ESM.pdf]

Fig. S1.

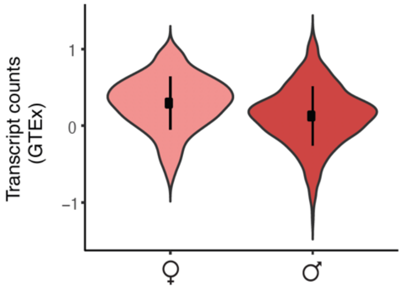

Violin plots of sex-biased gene expression in NLRP2. Expression in blood was quantified as transcript counts (GTEx).

Fig. S2.

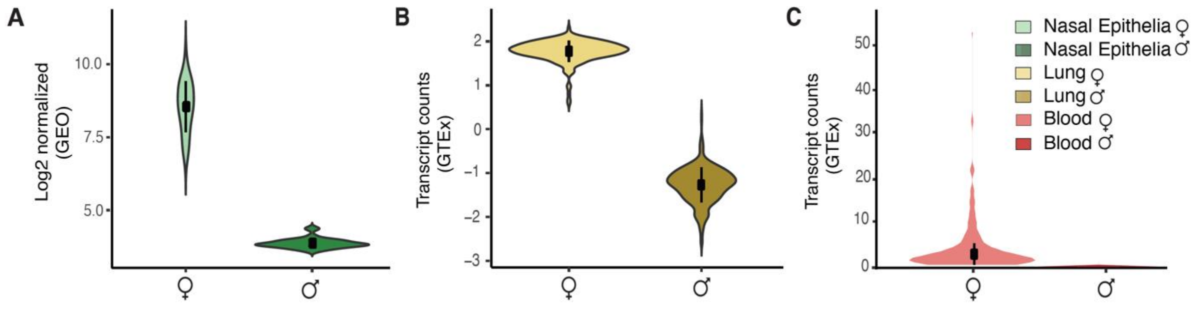

Violin plots of sex-biased gene expression in XIST. **(A)** Expression in nasal epithelia was measured as log2 normalized values (GEO). (**B)** Expression in lung was quantified as transcript counts (GTEx). (**C)** Expression in blood was quantified as transcript counts (GTEx).

Fig. S3.

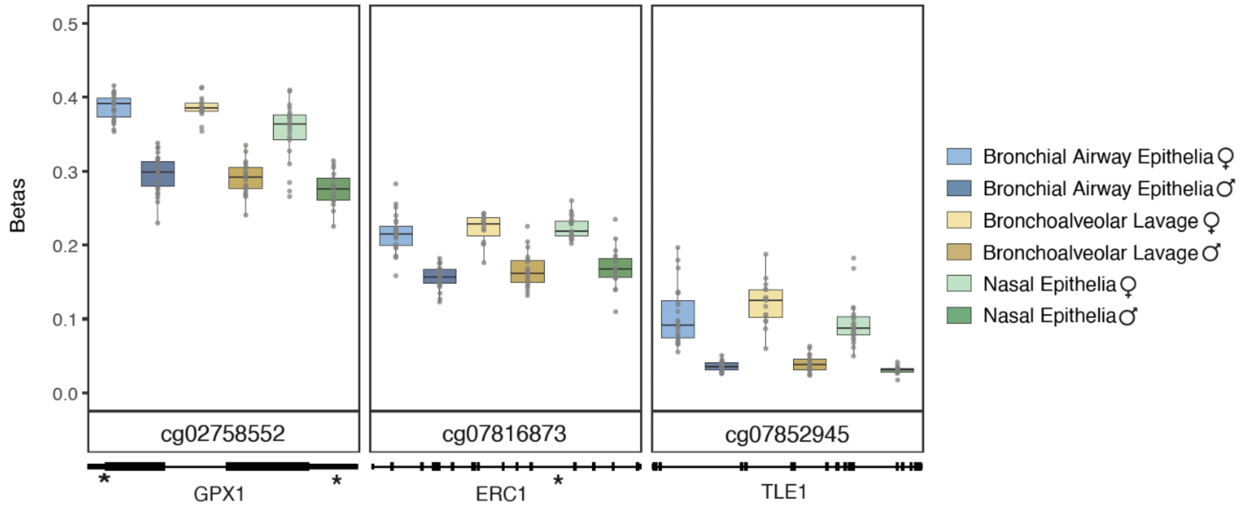

Box plots of the three validated CpG sites (GPX1, ERC1 and TLE1) differentially methylated by sex in three tissues from the repeated exposure dataset. Unadjusted DNA methylation values (β) were plotted on the y-axis with CpG sites on the x-axis. Genomic positions of the CpG sites are indicated below the respective plots.

Fig. S4.

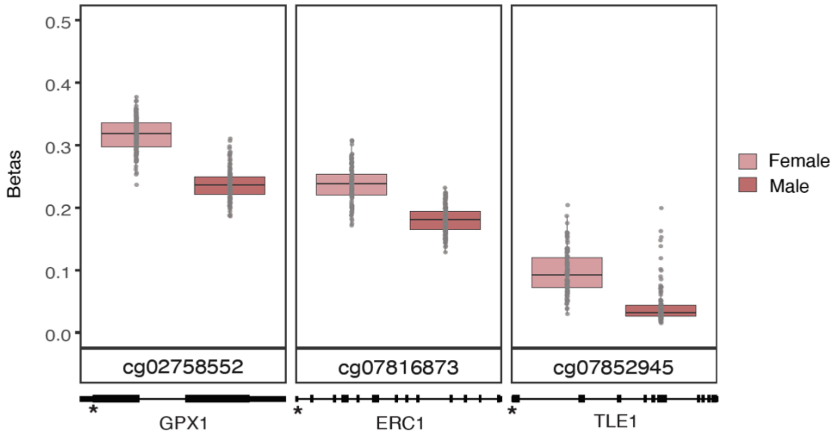

Box plots of the three validated CpG sites (GPX1, ERC1 and TLE1) differentially methylated by sex in blood. Unadjusted DNA methylation values (β) were plotted on the y-axis against the CpG sites on the x-axis, with genomic locations of the CpG sites plotted below the respective plots.

Fig. S5.

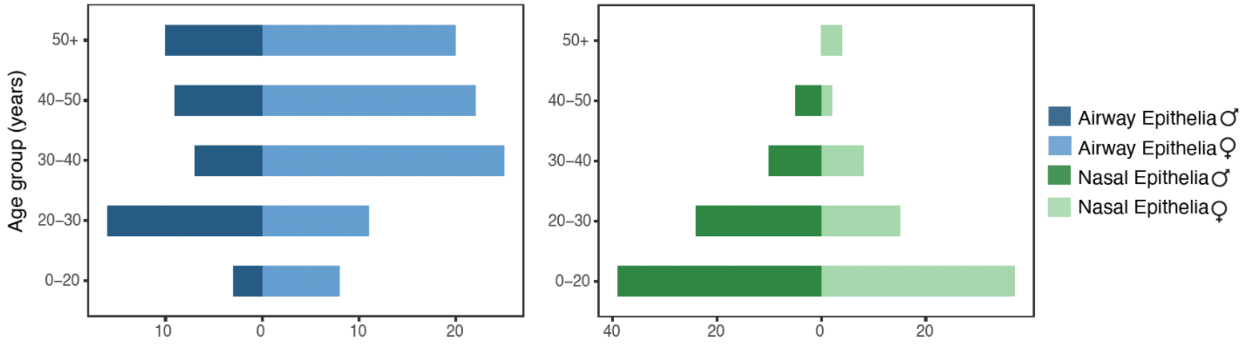

Bar plots of age distributions in airway epithelia and nasal epithelia. Age group in years is indicated on the y-axis while the x-axis represents the number of males and females.

Fig. S6.

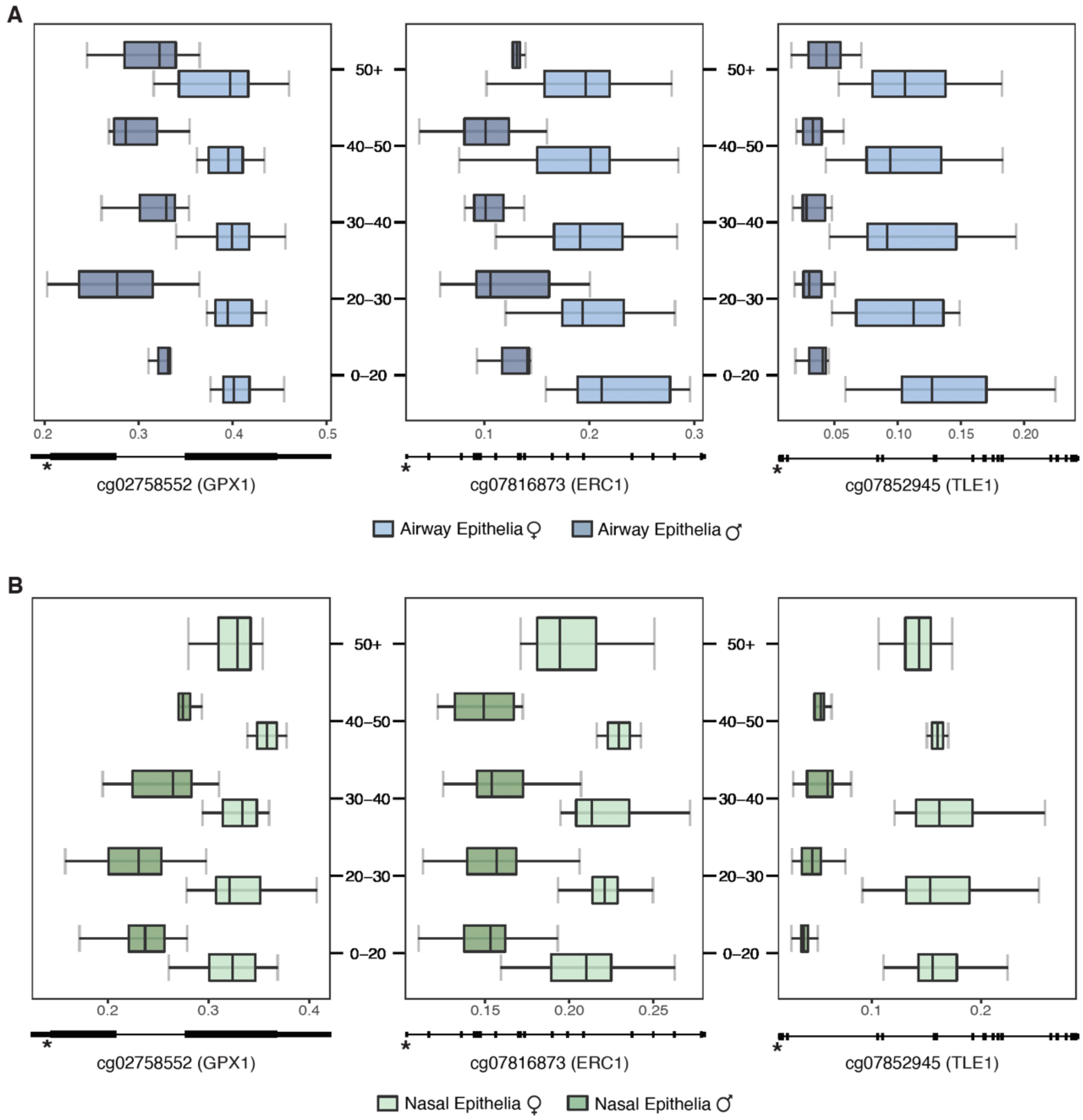

Box plots of the three validated CpG sites (GPX1, ERC1 and TLE1) differentially methylated by sex across the age groups in airway epithelia and nasal epithelia. Unadjusted DNA methylation values (β) were plotted on the x-axis with age groups in years on the y-axis. Genomic positions of the CpG sites are represented below the respective plots.

Fig. S7.

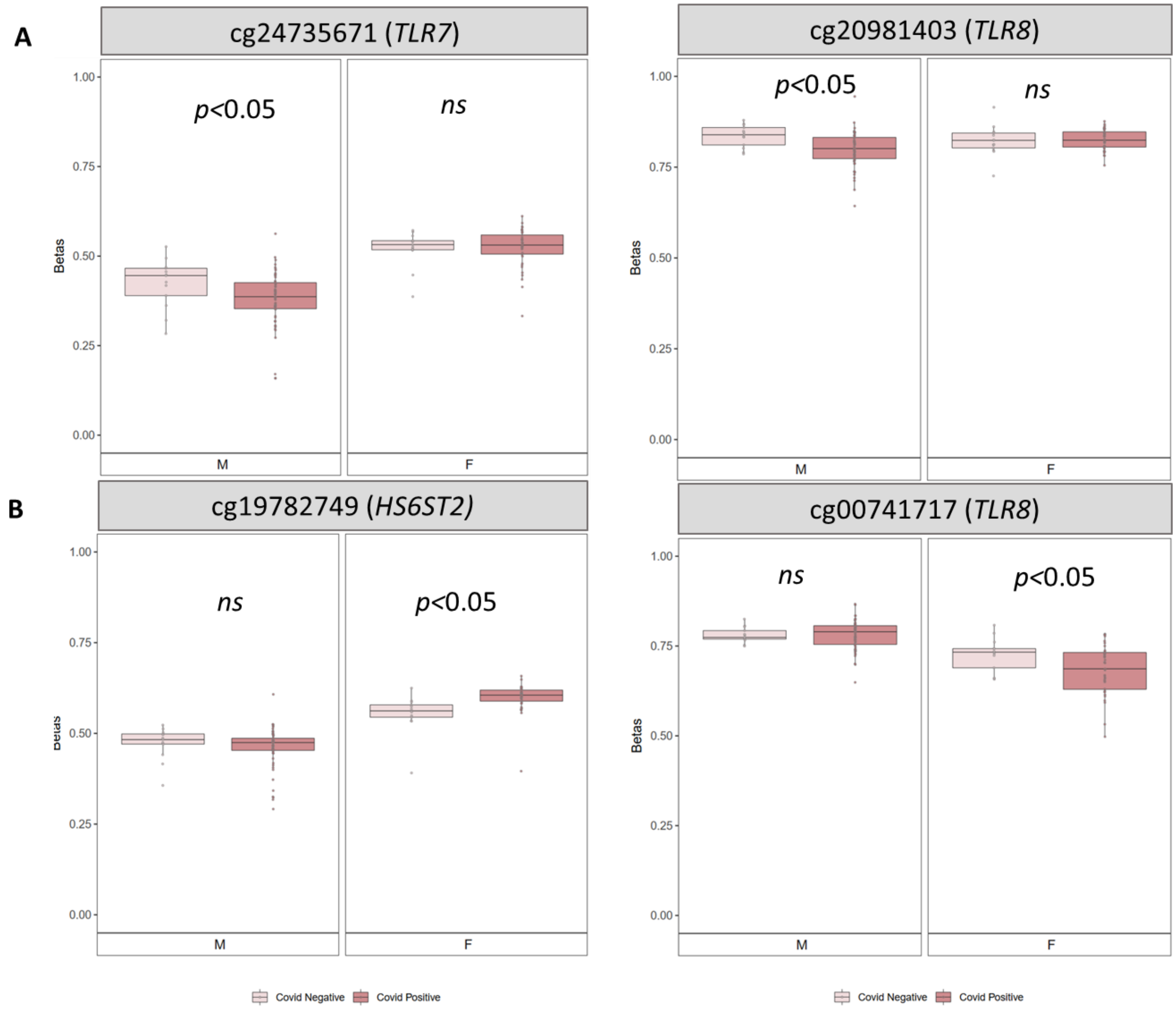

Box plots of the CpG sites that are differentially methylated by COVID-19 status in males (A) and in females (B). Unadjusted DNA methylation values (β) were plotted on the y-axis and the box plots are colored by COVID-19 status.
